# Evolutionary Patterns of Codon Usage in Major Lineages of Porcine Reproductive and Respiratory Syndrome Virus in China

**DOI:** 10.3390/v13061044

**Published:** 2021-05-31

**Authors:** Weixin Wu, Xinna Ge, Yongning Zhang, Jun Han, Xin Guo, Lei Zhou, Hanchun Yang

**Affiliations:** Key Laboratory of Animal Epidemiology of Ministry of Agriculture and Rural Affairs, College of Veterinary Medicine, Agricultural University, Beijing 100193, China; werson@cau.edu.cn (W.W.); gexn@cau.edu.cn (X.G.); zhangyongning@cau.edu.cn (Y.Z.); hanx0158@cau.edu.cn (J.H.); guoxin@cau.edu.cn (X.G.)

**Keywords:** porcine reproductive and respiratory syndrome virus (PRRSV), lineages, evolutionary analysis, codon bias, codon pair bias, host adaptability, virus attenuation

## Abstract

Porcine reproductive and respiratory syndrome virus (PRRSV) is economically important and characterized by its extensive variation. The codon usage patterns and their influence on viral evolution and host adaptation among different PRRSV strains remain largely unknown. Here, the codon usage of ORF5 genes from lineages 1, 3, 5, and 8, and MLV strains of type 2 PRRSV in China was analyzed. A compositional property analysis of ORF5 genes revealed that nucleotide C is most frequently used at the third position of codons, accompanied by rich GC3s. The effective number of codon (ENC) and codon pair bias (CPB) values indicate that all ORF5 genes have low codon bias and the differences in CPB scores among four lineages are almost not significant. When compared with host codon usage patterns, lineage 1 strains show higher CAI and SiD values, with a high similarity to pig, which might relate to its predominant epidemic propensity in the field. The CAI, RCDI, and SiD values of ORF5 genes from different passages of MLV JXA1R indicate no relation between attenuation and CPB or codon adaptation decrease during serial passage on non-host cells. These findings provide a novel way of understanding the PRRSV’s evolution, related to viral survival, host adaptation, and virulence.

## 1. Introduction

Porcine reproductive and respiratory syndrome virus (PRRSV) is an enveloped, single-stranded positive-sense RNA (+ssRNA) virus, which is classified into the genus *Porartevirus* of the family *Arteriviridae* in the order *Nidovirales* [1,2,3]. It is the etiological agent of porcine reproductive and respiratory syndrome (PRRS), which is clinically characterized by reproductive failure in sows, including abortion and elevated fetal losses, as well as respiratory disorders in pigs of all ages, leading to elevated mortality and poor growth performance, especially in weaning and nursery herds [4]. In the late 1980s, PRRSV first emerged as a “mystery” disease progressing through pig farms in both Europe and North America. Two prototype strains, Lelystad virus (LV) (European type, or type 1) and VR-2332 (North American type, or type 2), representing two genotypes of PRRSV with obvious genetic and antigenic differences, were first identified in Europe in 1991 and in the United States in 1992 [1,5]. The PRRSV genome is approximately 15 kb that carries at least 12 identified open reading frames (ORFs), in which ORF5 exhibits marked genetic variation. ORF5, the coding region of major glycoprotein GP5, was widely used as the target of evolutionary analysis. Based on the phylogenetic relationship of ORF5, type 2 PRRSV strains can be divided into nine distinct lineages [6].

China is the largest pork-producing country in the world, where PRRSV shows great genomic diversity in the field. The initial outbreak of PRRS in mainland China was recorded at the end of 1995. In 2006, an epidemic of atypical PRRS caused by the emerging highly pathogenic PRRSV (HP-PRRSV) was reported, resulting in huge economic losses to the pig industry [7]. Since 2011, NADC30-like strains, originally from the NADC30 virus isolated in the United States in 2008, have become epidemic in China [8]. The NADC30-like viruses belong to lineage 1 of type 2 PRRSV and they share lower nucleotide identities with previous representative PRRSV strains in China, including CH1a, BJ-4, HB-1(sh)/2002, HB-2(sh)/2002, and JXwn06 [8]. Several vaccination-challenge experiments have indicated that NADC30-like viruses are moderately virulent in piglets and most commercial PRRS vaccines only confer limited cross-protection against them [9,10]. According to a previous epidemiology investigation, most PRRSV field strains circulating in China belong to lineage 1, 3, 5, and 8 of the type 2 virus [11]. Soon after the outbreak of HP-PRRS in 2006, the lineage 8 strains had become the predominant virus in the field. Recently, the proportions of both lineage 1 and lineage 3 strains have been increasing, and lineage 1 is currently the predominant one [12]. However, the reasons for it being the predominant strain are still unclear. As an RNA virus, PRRSV has evolved at a high evolutionary rate, attributed to its low replication fidelity and strains’ recombination. Meanwhile, the selective pressure from hosts can further drive PRRSV evolution to adapt to the host [13].

It has been proved that synonymous codons are not chosen equally and randomly either within or between genomes [14,15], which is termed codon bias. The evolution of codon bias is affected by many factors, including natural selection, mutation pressure, genetic drift, and GC content [13,16,17]. Thus, according to codon usage analysis, the basic features of molecular evolution could be revealed [18]. The viral codon usage might be influenced by its host, as the viral replication needs synthetic machinery and metabolism from host cells [19]. Comparing viral codon usage patterns to those of its specific hosts helps us better understand the fitness and escape adaptations that take place during virus evolution [20]. The extent of codon usage bias between the virus and its host has been experimentally demonstrated to affect viral protein synthesis efficiency, replicative fitness, virulence, and even virus survival [21]. Although the previous analysis of synonymous codon usage in PRRSV has indicated that the synonymous codon usage patterns in different ORFs of PRRSV are different and genetically conserved [22], knowledge about codon usage among different lineages of PRRSV and how evolution influences them have not been reported yet. Consequently, 463 ORF5 sequences presenting different lineages of PRRSV were collected for phylogenetic and codon usage analysis to elucidate the similarities and differences of nucleotide composition, dinucleotide abundance, codon usage, codon pair bias, and host adaptation among different lineages during viral evolution. Furthermore, PRRSV field isolates can be attenuated as vaccine candidates, by serially passaging in MARC-145 cells, a subclone of African green monkey kidney epithelial cell line MA104. To further confirm if PRRSV attenuation is related to codon pair bias and whether codon pair bias changes to adapt non-porcine host cells, the JXA1 (or JXA1R, as it derived MLV) sequences from different passages were also analyzed for comparison with African green monkey’s and pig’s genomic codon usage datasets [23,24]. Our study not only provides detailed information on codon usage in PRRSV ORF5 genes, but also shows a novel approach to investigating PRRSV evolution, adaptation, and attenuation.

## 2. Materials and Methods

### 2.1. Data Collection

The sequences of 464 type 2 PRRSV field strains from China and different passages of HP-PRRSV JXA1 (or JXA1R as it derived MLV), together with 15 reference strains including both type 1 and type 2 prototype strain Lelystad virus and VR-2332, were retrieved from the GenBank nucleotide database recorded up to December 2020.

### 2.2. Recombination Detection and Phylogenetic Analysis

To investigate the codon usage bias of PRRSV ORF5 among different lineages, the sequences from the collected strains mentioned above were first aligned by using ClustalW in DNASTAR (version 11.1.0.54). Potential recombination events in ORF5 were identified using the recombination detecting program RDP4 (version 4.101) [25]. Recombination analysis of the aligned sequences was performed with default configuration using seven different algorithms (RDP, GENECONV, Chimaera, MaxChi, BootScan, 3Seq, and SiSca). A Bonferroni-corrected P-value cutoff of 0.05 was applied throughout the analysis. To avoid false-positive results, only recombination events supported by over four different methods were considered. The phylogenetic analysis was conducted by the Maximum Likelihood (ML) method in MEGA-X (version 10.2.2), using the Kimura 2-parameter model according to the Bayesian Information Criterion (BIC). The bootstrap value was set to 1000. The phylogenetic tree was viewed in iTOL (version 5.7) (https://itol.embl.de/) (accessed on 21 April 2021).

### 2.3. Nucleotide Composition

The nucleotide composition of PRRSV ORF5, including the frequencies of nucleotides (%A, %C, %U, and %G); the frequencies of nucleotides at the third position (%A3, %C3, %U3, and %G3); the GC contents (%G+C); the GC contents at the first (GC1s), second (GC2s), and third (GC3s) codon positions; and the frequency means of GC1s and GC2s (GC12s), were computed using CAIcal [26]. The frequencies of nucleotides at the third positions in the synonymous codons (A3s, C3s, U3s, and G3s) were computed by CodonW software (version 1.4.2) (http://codonw.sourceforge.net/) (accessed on 5 January 2021).

### 2.4. Dinucleotide Relative Abundance Analysis

Dinucleotide relative abundances effectively represent the contrasts between the observed dinucleotide frequencies and those expected from the component nucleotide frequencies. They were computed by using the following equation [27]:

(1) $$\rho_{xy}=\frac{f_{xy}}{f_{x}f_{y}}$$

where $f_{x}$ and $f_{y}$ denote the frequencies of the nucleotide x and y, respectively, and $f_{xy}$ is the frequency of the dinucleotide xy in the sequences under consideration. Relative abundance values $\rho_{xy}<0.78$ indicate that dinucleotide is underrepresented, while $\rho_{xy}>1.23$ indicates that dinucleotide is overrepresented [27]. The dinucleotide frequencies were computed by CodonW.

### 2.5. Codon Vias Analysis

#### 2.5.1. Effective Number of Codons Analysis

The effective number of codons (*ENC*) can be calculated from codon usage data alone, and it is independent of gene length and amino acid (aa) composition [28]. *ENC* can take values from 20, in the case of extreme bias where only one codon is exclusively used for each amino acid, to 61 when the use of alternative synonymous codons is equally likely. Consequently, it provides an intuitively meaningful measure of the extent of codon preference in a gene. *ENC* values were computed by using the following equation [28]:

(2) $$ENC=2+\frac{9}{\bar{F_{2}}}+\frac{1}{\bar{F_{3}}}+\frac{5}{\bar{F_{4}}}+\frac{3}{\bar{F_{6}}}$$

where $\bar{F_{i}}$ is the average, and $F_{i}$ is the i-fold degenerate amino acid family $F_{i}$ for each amino acid is calculated as follows [28]:

(3) $$F_{i}=\frac{n\sum_{j=1}^{i}{\left(\frac{n_{j}}{n}\right)}^{2}-1}{n-1}$$

where n is the total number of observed codons for that amino acid, and $n_{j}$ is the total number of observed jth codons for that amino acid. The *ENC* values were computed by the coRdon package (version 1.8.0) (https://github.com/BioinfoHR/coRdon) (accessed on 5 January 2021) of R (version 4.0.3) (https://www.r-project.org/) (accessed on 5 January 2021).

#### 2.5.2. Relative Synonymous Codon Usage Analysis

Relative synonymous codon usage (*RSCU*) is used to examine synonymous codon usage without the confounding influence of the amino acid composition of different gene products [29]. The observed numbers of codons were converted to relative synonymous codon usage values using the following equation [29]:

(4) $$RSCU_{ij}=\frac{X_{ij}}{\frac{1}{n_{i}}\sum_{j=1}^{n_{i}}x_{ij}}$$

where $X_{ij}$ is the number of occurrences of the jth codon for the ith amino acid, which has $n_{i}$ synonymous codons. In short, *RSCU* is the observed number of occurrences divided by the number that would be expected if synonymous codons were used uniformly. An *RSCU* value = 1.0 indicates no codon usage bias, and an *RSCU* value > 1.0 represents positive bias, while an *RSCU* value < 1.0 represents negative bias. Besides, codons with *RSCU* values > 1.6 will be regarded as overrepresented, while codons with *RSCU* values < 0.6 will be said to be underrepresented [30]. The *RSCU* values were computed by the seqinr package (version 4.2.4) (http://seqinr.r-forge.r-project.org/) (accessed on 5 January 2021) of R.

#### 2.5.3. Principal Component Analysis

Principal component analysis (PCA) is used to summarize the distance matrix, which records distances between each combination of samples [31], and it is used to identify the correlations between variables and samples. In our study, each sequence was represented as a 59-dimensional vector to reduce the effect of the amino acid composition on codon usage and the RSCU value of each codon corresponds to each dimension, while codons UGG and AUG and the three termination codons were excluded from this analysis. The 59-dimensional vector was transformed into two major axes, and we plotted the first two principal components along the X and Y-axis. The PCA analysis was performed by the factoextra package (version 1.0.7) (http://www.sthda.com/english/rpkgs/factoextra) (accessed on 5 January 2021) of R.

### 2.6. Codon Pair Bias Analysis

Similarly, but independently of codon bias, the juxtaposition of codons in ORFs does not appear to be randomly distributed either [32], and these preferences are typically referred to as codon pair bias. An algorithm has been developed to quantify codon pair bias [33]. For each of the 3721 possible codon pairs, excluding stop codon pairs, the codon pair score (*CPS*) is defined as the natural log of the observed ratio over the expected number of occurrences of each codon pair’s overall coding regions. It can be calculated by using the following equation [33]:

(5) $$CPS=ln\left(\frac{F\left(AB\right)}{\frac{F\left(A\right)\times F\left(B\right)}{F\left(X\right)\times F\left(Y\right)}\times F\left(XY\right)}\right)$$

where the codon pair AB encodes for amino acid pair XY, and F denotes frequency (number of occurrences). The *CPS* value for a given pair determines whether the pair is over-represented (+) or under-represented (−). With the calculated *CPSs*, we can further calculate the codon pair bias (*CPB*) score as follows [33]:

(6) $$CPB=\overset{k}{\underset{i=1}{\sum }}\frac{CPS_{i}}{k-1}$$

where k indicates how many kinds of codon pairs there are. The *CPB* score has already been used for virus attenuation through deoptimization [33,34,35]. Theoretically, a decreased *CPB* score is associated with the inefficiency of the viral gene translation in the host, which results in attenuation of viral replication [36]. The *CPB* scores were computed by the *CPBias* package (version 1.0) (https://github.com/alex-sbu/CPBias/) (accessed on 5 January 2021) of R.

### 2.7. Codon Usage Comparison Between Viruses and Hosts

#### 2.7.1. Codon Adaptation Index Analysis

The codon adaptation index (CAI) is a measure of directional synonymous codon usage bias, which is useful for predicting the level of gene expression, assessing the adaptation of viral genes to their hosts, and making comparisons between codon usage in different organisms [37]. CAI values range from 0 to 1, and the sequences with higher CAIs indicate stronger adaptability to the host. The CAI values were computed by CAIcal [26]. The reference datasets of synonymous codon usage patterns of the pig (*Sus scrofa*), which is the host of PRRSV, were downloaded from the Codon and Codon Pair Usage Tables database (CoCoPUTs) (updated in January 2020) [38] and used for analyzing PRRSV ORF5 genes. The reference datasets for analyzing JXA1-attenuated strains were downloaded from the Codon Usage Database (updated in June 2007) [39], which contains the synonymous codon usage patterns of the pig (*Sus scrofa*) and the African green monkey (*Chlorocebus sabaeus*).

#### 2.7.2. Relative Codon Deoptimization Index Analysis

Relative codon deoptimization index (RCDI) is a comparative measure against the general codon distribution. It was first used for analyzing the human genome [40] and then used in other species [41,42,43]. RCDI provides an estimate of the efficiency of viral gene translation in a specific host. The RCDI values, higher than 1, indicate the deoptimization of the codon usage patterns of the virus from that of its host(s), but if the codon usages of a pathogen and its host(s) are similar, the RCDI value is close to 1 [44]. The RCDI values were computed by CAIcal [26].

#### 2.7.3. Similarity Index Analysis

The similarity index (*SiD*) was used to evaluate the potential role of the overall codon usage pattern of the host in the formation of the overall codon usage of viruses, which can be calculated using the following equation [45]:

(7) $$R\left(A,B\right)=\frac{\sum_{i=1}^{59}a_{i}\times b_{i}}{\sqrt[{2}]{\sum_{i=1}^{59}a_{i}^{2}\times \sum_{i=1}^{59}b_{i}^{2}}}$$

(8) $$SiD=D\left(A,B\right)=\frac{1-R\left(A,B\right)}{2}$$

where $a_{i}$ is the *RSCU* value for a specific codon in 59 synonymous codons of the virus, and $b_{i}$ is the *RSCU* value for the same codon of the host. The *SiD* values range from 0 to 1.0, with a higher *SiD* value indicating a more influential role.

### 2.8. Statistical Analysis

Because the values of *CAI*, *RCDI*, *SiD*, and *CPB* score were not strictly normally distributed and the lineages had unequal variances [21], the non-parametric Kruskal–Wallis test and Bonferroni-corrected Dunn’s multiple comparison test were used to investigate the statistically significant differences of *CAI*, *RCDI*, *SiD*, and *CPB* scores in our study. Significant relationships are shown in box-plots with an extremely significant relationship (***) of *p* ≤ 0.001, a highly significant relationship (**) of 0.001 < *p* ≤ 0.01, a significant relationship (*) of 0.01 < *p* ≤ 0.05, and no significant relationship (NS.) of 0.05 < *p*. Statistical analysis and box-plot generation were performed using the ggpubr package (version 0.4.0) (https://rpkgs.datanovia.com/ggpubr/) (accessed on 5 January 2021) of R.

## 3. Results

### 3.1. Recombination and Phylogenetic Analysis

**Figure 1:**
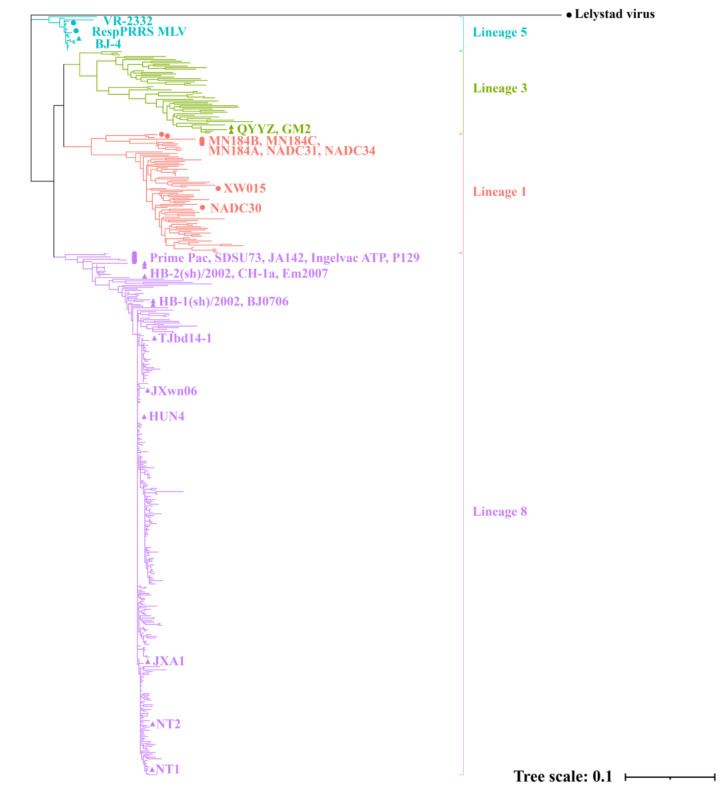
Phylogenetic tree based on PRRSV ORF5 genes. Type 1 Lelystad virus is set as outgroup reference, and type 2 lineage 1, 3, 5, and 8 are represented in orange, green, blue, and purple, respectively. Reference strains are marked as a triangle (for Chinese strains) and circle (for reference strains from other countries).

One PRRSV ORF5 sequence was found to have a potential recombination signal. After removing the recombinant sequence, the remaining 463 sequences were used for further analysis. A phylogenetic tree (Figure 1) was constructed based on ORF5 sequences from 463 Chinese PRRSV strains and 15 referent sequences. The type 1 prototype strain Lelystad virus was set as an outgroup reference. Phylogenetic analysis showed that selected PRRSV strains can be clustered into four different lineages among the nine lineages of type 2 PRRSV [6]: Lineage 1 (*n* = 68, first found in China in 2011), lineage 3 (*n* = 52, first found in China in 2009), lineage 5 (*n* = 20, first found in mainland China in 1996), and lineage 8 (*n* = 323, first found in mainland China in 1995). Some representative strains were selected from each lineage to show their strain name. The detailed sequence information (accession number, strain name, location, lineage, and properties) for PRRSV sequences can be found in Supplementary Materials (Supplementary Table S1).

### 3.2. Nucleotide Composition

The results in Table 1 show that nucleotide U (0.296 ± 0.005 in all) is the most frequent of all the strains, while the mean value of %C3 (0.320 ± 0.012 in all) is the highest at the third codon position, followed by %U3 (0.290 ± 0.012 in all), %G3 (0.268 ± 0.014 in all), and %A3 (0.122 ± 0.011 in all). The nucleotides at the third position of synonymous codons show similar composition patterns among these four lineages. %G + C (0.501 ± 0.007 in all) was at a middle level among GC1s (0.469 ± 0.012 in all), GC2s (0.445 ± 0.009 in all), and GC3s (0.588 ± 0.016 in all), in which GC3s are the highest, and lineage 8 has the highest GC3s value (0.592 ± 0.010). Lineage 5 and 8 have higher GC12s values (0.466 ± 0.003 and 0.459 ± 0.005) than lineage 1 and 3 (0.447 ± 0.006 and 0.451 ± 0.007).

### 3.3. Dinucleotide Relative Abundance Analysis

**Figure 2:**
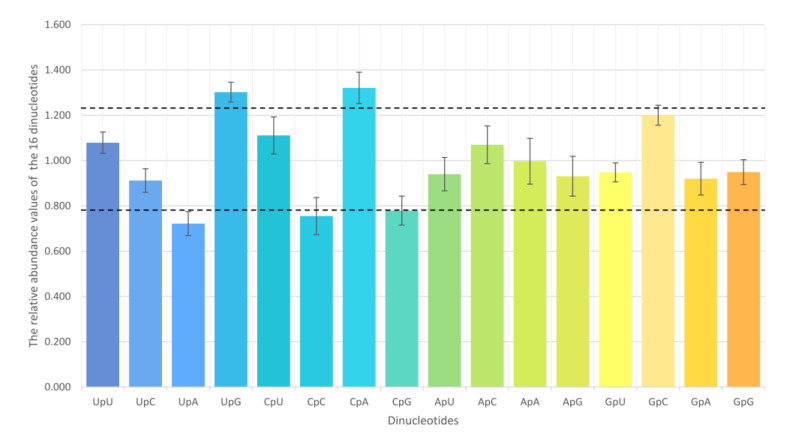
Dinucleotide abundance of ORF5 genes from Chinese PRRSV strains. Dinucleotides are regarded as underrepresented or overrepresented if the relative abundance values are below 0.78 or above 1.23 (dashed lines), respectively.

Analysis of the 16 dinucleotides shows that no dinucleotide frequency is equal to the expected value, indicating that no dinucleotide is randomly used (Figure 2). The dinucleotides UpG (1.302 ± 0.045 in all) and CpA (1.321 ± 0.071 in all) are both overrepresented (value > 1.23) in each lineage, and UpA is underrepresented in lineages 3 (0.716 ± 0.089), 5 (0.723 ± 0.020), and 8 (0.710 ± 0.032) (Table 2). UpA is not considered as underrepresented in lineage 1 (0.783 ± 0.060), because it is close to 0.78 (the boundary of underrepresented). Interestingly, the dinucleotides CpC (0.712 ± 0.033) and CpG (0.749 ± 0.030) are significantly underrepresented in lineage 8, compared with those in lineages 1, 3, and 5. That made the mean values of CpC (0.755 ± 0.082 in all) and CpG (0.779 ± 0.064 in all) are classified as underrepresented. Furthermore, the dinucleotide ApG in lineage 1 (0.765 ± 0.055) is also significantly underrepresented compared to those in other lineages.

### 3.4. Codon Bias and Codon Pair Bias Analysis

The ENC values range from 20 to 61. In this study, lineage 5 has the highest value (60.797 ± 0.624), followed by lineage 8 (59.645 ± 1.239), lineage 3 (59.618 ± 2.002), and lineage 1 (59.653 ± 1.643) (Table 1). That means PRRSV ORF5 genes have extremely low codon usage bias, especially in lineage 5.

Although the ENC values indicate that up to 60 codons are used in the PRRSV genome, the usage frequency for each codon is different. *RSCU* analysis shows that some codons are overrepresented (*RSCU* > 1.6) or underrepresented (*RSCU* < 0.6) in four lineages (Supplementary Table S2). Codons AGC (Ser) (1.801 ± 0.242 in all) and ACC (Thr) (2.062 ± 0.205 in all) are overrepresented in all four lineages, and UUG (Leu) is overrepresented in the three lineages except lineage 8 (1.706 ± 0.301 in all). What is noteworthy is that codon CCC (Pro) is overrepresented in lineage 3 (1.602 ± 0.713) and lineage 8 (1.978 ±0.305), while it is low in lineage 5 (0.780 ± 0.194) and underrepresented in lineage 1 (0.564 ± 0.346). UUA (Leu) (0.396 ± 0.150 in all), AUA (Ile) (0.137 ± 0.215 in all), GUA (Val) (0.126 ± 0.205 in all), ACA (Thr) (0.498 ± 0.130 in all), ACG (Thr) (0.315 ± 0.122 in all), and GGA (Gly) (0.390 ± 0.231 in all) are underrepresented in all lineages, which leads to the low value of A3s. In addition, CUA (Leu) and AGU (Ser) in lineage 5 show relatively higher RSCU values than those in other lineages, and that is similar for CCA (Pro) in lineage 1. The results of RSCU are correlated with the overrepresented dinucleotide CpA, and UpG, as well as the underrepresented UpA.

**Figure 3:**
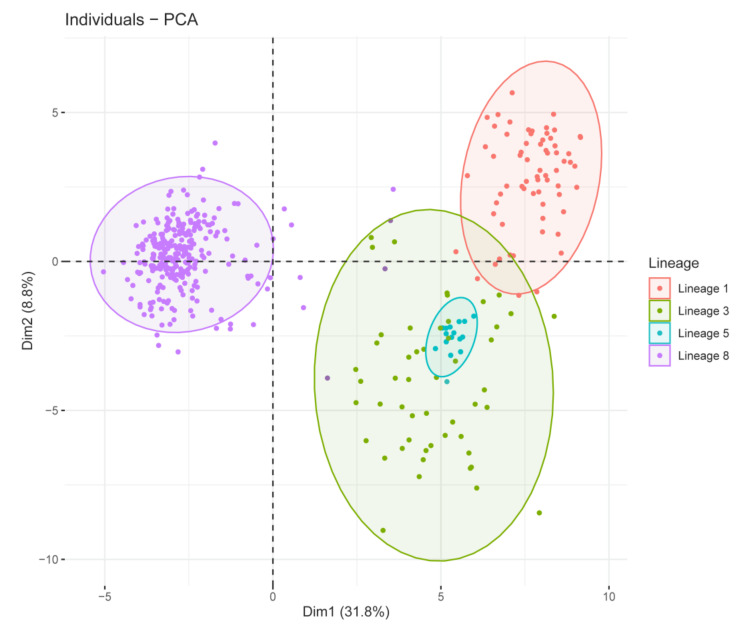
PCA-plot based on ORF5 genes’ RSCU value from different lineages of Chinese PRRSV strains.

In PCA analysis based on *RSCU* values of the 59 synonymous codons, the first two principal axes of PRRSV ORF5 genes accounted for 31.8% and 8.8% of the synonymous codon usage, and we explored the distribution of each lineage based on the first two axes (Figure 3). Intriguingly, we found that lineage 5 completely overlapped with lineage 3, and there were several overlaps between lineage 3 and lineage 1, which indicated that the codon usage patterns of lineage 5 are very similar to lineage 3, and lineage 3 is partially similar to lineage 1, while for lineage 8, the separate part has its usage patterns.

**Figure 4:**
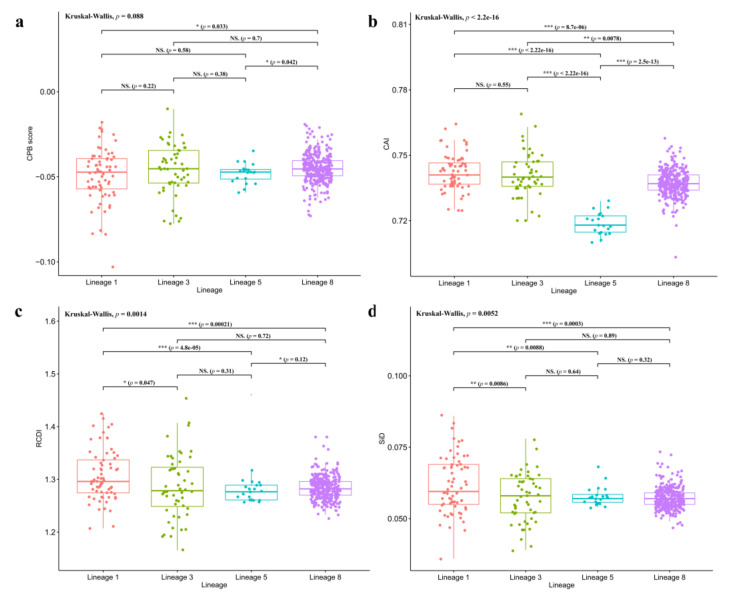
CPB scores (**a**), *CAI* (**b**), *RCDI* (**c**), and *SiD* (**d**) box-plots of ORF5 genes from different lineages of Chinese PRRSV strains. The asterisk indicates a significant difference between labeled groups (NS. 0.05 < *p*; * 0.01 < *p* ≤ 0.05; ** 0.001 < *p* ≤ 0.01; *** *p* ≤ 0.001).

Codon pair bias (*CPB*) scores of Chinese PRRSV strains were also calculated in this study (Figure 4a). The mean value of lineage 8 (−0.045 ± 0.009) is slightly significantly higher than that of lineage 1 (−0.049 ± 0.016) and lineage 5 (−0.048 ± 0.006) (*p* = 0.038 and *p* = 0.03, respectively), while the mean value of lineage 3 (−0.046 ± 0.015) is between them. It has been proved that a PRRSV ORF5 CPB score decreasing from −0.049 to −0.354 could confer attenuation of virulence in PRRSV [46]. Thus, we cannot easily assert that the result is related to the prevalence or virulence of lineage 8.

### 3.5. Codon Usage Comparison Between Virus and Host

*CAI* (Figure 4b), *RCDI* (Figure 4c), and *SiD* (Figure 4d) were used to compare the codon usage patterns between PRRSV and pig in this study, by using the Codon and Codon Pair Usage Tables database (CoCoPUTs) updated in January 2020 [38]. The *CAI* values of lineage 1 and lineage 3 are significantly higher than that of lineage 8 (*p* ≤ 0.001 and 0.001 < *p* ≤ 0.01, respectively, Dunn’s test), and they are all much higher than that of lineage 5 (*p* ≤ 0.001, Dunn’s test). Considering that the higher values might indicate higher gene expression potential and adaptability, it might contribute to increased circulating of lineage 1 and 3 strains in fields of China recently.

*CAI* is a measure of codon usage adaptation to the most used synonymous codons of the reference genome and is commonly used to predict gene expression efficiency. On the other hand, the *RCDI* is used to assess whether the codon usage of a gene is similar to that of the reference genome [47]. For *RCDI*, lineage 1 is significantly higher than lineage 8 and 5 (*p* ≤ 0.001, Dunn’s test), and lineage 5 is significantly lower than lineage 8 (0.01 < *p* ≤ 0.05, Dunn’s test). It has been proved in poliovirus that viruses with higher *RCDI* values are not well adapted to their host [40]. However, lineage 1 has been proved to be the most circulating lineage in fields, which is contrary to the *RCDI* analysis result.

For *SiD*, lineage 1 has significantly higher values than lineages 3 and 5 (0.001 < *p* ≤ 0.01, Dunn’s test), and is much higher than lineage 8 (*p* ≤ 0.001, Dunn’s test), while among other lineages, there was no statistically significant difference, indicating that the pigs had a significantly deeper influence on lineage 1 than others.

### 3.6. JXA1-Attenuated Strain Analysis

**Figure 5:**
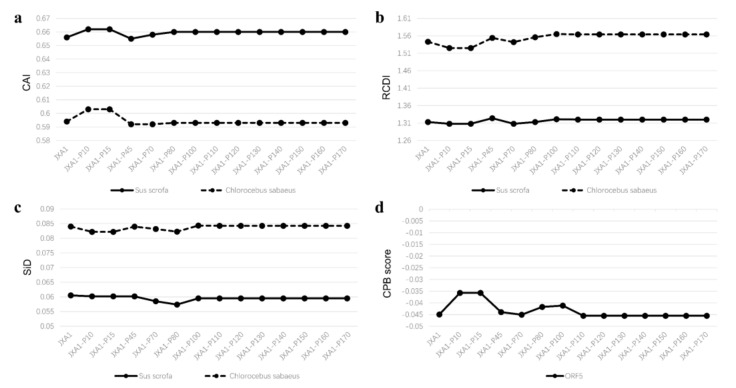
*CAI* (**a**), *RCDI* (**b**), *SiD* (**c**), and *CPB* scores (**d**) of ORF5 genes from different passages of JXA1 during attenuation.

To investigate whether the attenuation of PRRSV MLV strains is related to *CPB* changes from its natural host pig to non-pig patterns (monkey original MARC-145 cells), codon adaptation analysis on attenuated HP-PRRSV JXA1 was applied. The *CAI* (Figure 5a), *RCDI* (Figure 5b), and *SiD* (Figure 5c) values of each passage were calculated referring to the codon usage database of the pig (*Sus scrofa*), as well as the African green monkey (*Chlorocebus sabaeus*), which was obtained from the Codon Usage Database (updated in June 2007) [39]. As the passages increased, the values of *CAI*, *RCDI*, and *SiD* showed no obvious unidirectional change, though little fluctuation could be observed. The *CPB* score also has a slight non-unidirectional float (Figure 5d). As mentioned above, PRRSV ORF5 *CPB* scores that fell by about 0.305 could attenuate the virulence of PRRSV [46], while *CPB* scores only dropped 0.0011 from passage 45 to 70 (from −0.04394 to −0.04505) in the present study. Furthermore, high *CAI* and low *RCDI* values, as found in the pig’s codon usage table, indicated that the virus is still more adaptive to pigs than African green monkeys. The African green monkey, however, with higher *SiD* values, influences JXA1 strains’ codon usage bias more than the pig. Overall, our study proved that the phenotypic modulation of JXA1-attenuated strains is not related to its codon pair bias or codon usage de-adaptation of its major glycoprotein to the host.

## 4. Discussion

PRRSV is an economically important pathogen characterized by its extensive genetic and antigenic variation among field strains. Because of its fast evolution as well as lack of efficient heterologous cross-protection, the introduction of variant strains can usually cause disease outbreaks in a PRRS stable herd. Thus, monitoring PRRSV genetic variation and exploring the mechanism of its evolution is very meaningful for PRRS control. Previous phylogenetic research on global-rang strains has shown that type 2 PRRSV could be clustered into nine different lineages, based on the ORF5 genes. Some lineages have caused epidemics in certain countries or regions and then become predominant during a period, but some others are found less in the field. To explore the evolutionary characterizations and host adaptability of PRRSV from different lineages in China, codon usage analysis was carried out here.

In previous studies, the codon usage patterns in different ORFs of PRRSV have been analyzed, and the nsp1 α, nsp9, and ORF5 have been used as the targets of codon deoptimization to attenuate the virus [22]; however, the differences of codon usage among different lineages have still not been reported. Thus, at the beginning of this study, many analysis strategies were considered and excluded, until ORF5 was finally set as the target gene. As the total size of conserved coding regions of the PRRSV genome is larger than that of other variant regions, it will “dilute” the variation of hypervariable nsp2 and ORF5, if all coding ORFs are counted. Meanwhile, there is the possibility that nsp2 coding regions might be from different lineages as the recombination, and the main object of this study is to compare codon usage among different lineages; thus, the strategy to combine nsp2 and ORF5 in analysis was also rejected. Before the phylogenetic analysis, the recombination event of ORF5 was initially analyzed to rule out the recombinant strains. However, only 1 of 464 strains was confirmed to be a recombinant virus by over four different algorithms in the detecting program RDP4. This is in agreement with our previous study that is based on 272 PRRSV genomes from China, during the years 2012–2017. In that study, 94 breakpoints were identified in 38 recombinant strains, among which only 3 breakpoints were identified in ORF5; however, there were 27 and 16 breakpoints identified in nsp2 and nsp9 coding regions, respectively (unpublished data).

For all the analyzed strains in this study, nucleotide U is the most abundant, and C is the most frequently used at the third position of codons, accompanied by rich GC content at the third position (GC3s) in the ORF5 genes. The increment on nucleotide U in RNA viruses over time might be linked to their adaptation and evolution in mammalian hosts [48].

According to our data, the mean ENC value of PRRSV (59.684 ± 1.415) is higher than that of other porcine viruses like porcine astrovirus (PAstV) (53.83 ± 1.902) [19], atypical porcine pestivirus (APPV) (54.832 ± 0.254) [21], Nipah virus (NiV) (51.57 ± 1.64) [41], and classical swine fever virus (CSFV) (51.85 ± 0.39) [49], which means that most codons are used by PRRSV. It is also worth noting that lineage 5 has the highest ENC values (lowest codon bias) compared to other lineages. There is a possibility that commercial MLVs from lineage 5 are widely used in the field, which might provide better protection against the virus from the same lineage, resulting in fewer epidemics in the field and withdrawing from the “evolutionary arena”. This is currently observed in both China and the United States, which might reduce the possibility of mutation to change its codon bias for adaptation.

Each lineage has formed specific codon usage patterns during evolution. The underrepresented and overrepresented results of codon *RSCU* values would be influenced by both GC content and the higher-order nucleic acid structures [29]. The simplest higher structures, dinucleotides, are often non-random in frequency, which plays an important role in codon bias. Previous studies on RNA viruses have shown that the marked dinucleotide CpG deficiency is a selective pressure contributing to codon usage bias [50,51,52]. This pressure helps them in escaping the host’s antiviral immune response, and the usage of CpG in +ssRNA viruses is greatly influenced by hosts’ CpG usage [48]. In this study, CpG is significantly underrepresented in lineage 8, compared with that in lineage 1, 3, and 5. The highly pathogenetic strains in lineage 8 might be connected to that. The relatively low abundance of UpA has also been commonly observed in other RNA viruses [52], as viruses can benefit from UpA deficiency in two possible ways. First, UpA is the RNA dinucleotide that is most susceptible to RNase activity, and it has been reported that ribonuclease L (RNase L), which can degrade RNA molecules and activate apoptotic pathways as a part of the vertebrate antiviral pathway, preferentially targets UpA or UpU sites in West Nile virus [27,53]. Next, UpA is the integral part in two out of three stop codons as well as in the transcriptional regulatory motifs [27,54]. Thus, the deficiency of dinucleotide UpA in PRRSV might be able to reduce the risk of nonsense mutations and minimize the chances of cleavage by RNase L.

Codon pair bias showed no efficient difference among the four lineages; hence, the virulence difference among lineages might not be the result of codon pair bias. A codon usage preference offers an evolutionary force driving the overall viral fitness during replication [55]. One study replaced native codons of PRRSV ORF5 genes with those more closely reflecting a preference of highly expressed mammalian genomes, resulting in a 6 to 11 times increase in expression efficiency [56]. That research greatly proved the importance of codon adaptation to hosts. In this study, the *CAI* and *SiD* values suggest that lineage 1 has higher gene expression potential than other lineages. Lineage 1 virus was more deeply influenced by pigs during evolution, which is consistent with their high prevalence in China and the United States. Nevertheless, the *RCDI* values were unexpectedly high in lineage 1. It is generally recognized that the fitness and virulence of a virus are commonly coupled phenomena and should be positively correlated. However, certain mutations/genetic changes may exist, which can break this fitness–virulence relationship owing to the complex virus–host interactions [41]. The high *RCDI* value may reflect the expression of a few genes during latency or maintenance of a low translation rate to achieve error-proof translation and correct folding of viral proteins [41]. Virulence studies from Khandia [41] and Furió [57] also showed unanticipated relations between virulence and RCDI values.

Serial passage in a heterologous host is a classic method of attenuating viruses, while many innovative methods are used to fasten viral attenuation. For example, engineering viral codons for more serine and leucine codons with nonsense mutation targets could generate stop mutations after a single nucleotide substitution, leading to viruses generating more stop mutations both in vitro and in vivo, accompanied by significant losses in viral fitness [58]. Furthermore, attenuation by codon pair deoptimization has been widely reported as a strategy for the rapid and highly efficacious attenuation of various RNA viruses, through increasing the number of codon pairs that are underrepresented in the protein-coding sequences of the host and creating unfavorable conditions for protein production, processing, or folding [33,35,36,59,60,61,62,63,64]. Meanwhile, many nucleotide and amino acid mutations have been reported to relate to increased virulence or attenuation of PRRSV, and our group has also performed some studies on the mechanism of pathogenicity changes through a reverse genetic operation. However, many identified mutation sites or genes were strain-specific and few conserved sites or genes were found. Thus, we wondered whether there was a possibility that the codon or codon pair usage might be changed when the PRRSV adapted the non-host cells to attenuation. However, the analysis conducted on JXA1-attenuated strains indicates no significant codon pair usage differences through passages on MARC-145 cells. Meanwhile, it is interesting that the African green monkey plays a more critical role in affecting JXA1′s codon usage pattern. This may indicate that although the JXA1 strain is more adaptive to pigs, the virus may have undergone some changes in codon usage patterns during passage in African green monkey kidney-derived cells. Besides ORF5, other coding ORFs of JXA1 were compared in codon pair bias; however, the results are similar to those of ORF5, so they are not shown here.

In summary, detailed information about the codon usage of PRRSV ORF5 genes from major lineages of China was systematically provided in this study. The codon usage pattern of different lineages of PRRSV reflects the evolutionary changes made to survive and adapt to hosts. Compared with viruses in the other three Chinese lineages, the lineage 1 strains show higher similarity in codon usage to pigs, with higher *CAI* and *SiD* values, which might relate to its host adaptation and predominance in the field. It is also first proved here that there is no relation between attenuation and codon pair bias or codon adaptation decrease during HP-PRRSV serial passaging on MARC-145 cells. These findings could provide a novel way of further understanding the PRRSV evolutionary changes related to viral survival, host adaptation, and virulence.

## Figures and Tables

**Table 1:** Properties of PRRSV ORF5 genes from Chinese strains analyzed in this study (mean value ± SD).

Categories	Lineage 1	Lineage 3	Lineage 5	Lineage 8	All
%A	0.214 ± 0.004	0.210 ± 0.006	0.203 ± 0.002	0.201 ± 0.003	0.204 ± 0.006
%C	0.236 ± 0.006	0.241 ± 0.007	0.244 ± 0.003	0.241 ± 0.003	0.240 ± 0.005
%U	0.296 ± 0.006	0.295 ± 0.010	0.294 ± 0.003	0.295 ± 0.003	0.296 ± 0.005
%G	0.254 ± 0.005	0.254 ± 0.007	0.259 ± 0.002	0.263 ± 0.003	0.260 ± 0.005
%A3	0.131 ± 0.008	0.135 ± 0.012	0.129 ± 0.004	0.118 ± 0.008	0.122 ± 0.011
%C3	0.318 ± 0.014	0.333 ± 0.021	0.324 ± 0.007	0.317 ± 0.006	0.320 ± 0.012
%U3	0.293 ± 0.014	0.283 ± 0.027	0.297 ± 0.005	0.290 ± 0.006	0.290 ± 0.012
%G3	0.258 ± 0.009	0.249 ± 0.015	0.250 ± 0.004	0.275 ± 0.008	0.268 ± 0.014
A3s	0.183 ± 0.011	0.186 ± 0.016	0.178 ± 0.005	0.163 ± 0.011	0.169 ± 0.015
C3s	0.365 ± 0.016	0.382 ± 0.025	0.372 ± 0.007	0.361 ± 0.007	0.365 ± 0.014
U3s	0.336 ± 0.015	0.324 ± 0.030	0.341 ± 0.007	0.330 ± 0.007	0.331 ± 0.014
G3s	0.323 ± 0.015	0.309 ± 0.020	0.303 ± 0.007	0.341 ± 0.013	0.333 ± 0.019
%G + C	0.490 ± 0.007	0.495 ± 0.011	0.502 ± 0.004	0.504 ± 0.004	0.501 ± 0.007
GC1s	0.452 ± 0.007	0.451 ± 0.010	0.481 ± 0.004	0.475 ± 0.006	0.469 ± 0.012
GC2s	0.442 ± 0.009	0.451 ± 0.011	0.451 ± 0.004	0.444 ± 0.008	0.445 ± 0.009
GC3s	0.576 ± 0.015	0.583 ± 0.031	0.574 ± 0.009	0.592 ± 0.010	0.588 ± 0.016
GC12s	0.447 ± 0.006	0.451 ± 0.007	0.466 ± 0.003	0.459 ± 0.005	0.457 ± 0.007
ENC	59.653 ± 1.643	59.618 ± 2.002	60.797 ± 0.624	59.645 ± 1.239	59.693 ± 1.406

**Table 2:** Dinucleotide relative abundance of PRRSV ORF5 genes from Chinese strains analyzed in this study (mean value ± SD).

Categories	Lineage 1	Lineage 3	Lineage 5	Lineage 8	All
UpU	1.073 ± 0.048	1.128 ± 0.086	1.053 ± 0.029	1.075 ± 0.032	1.079 ± 0.047
UpC	0.862 ± 0.045	0.965 ± 0.088	0.962 ± 0.023	0.911 ± 0.032	0.912 ± 0.052
UpA	0.783 ± 0.060	0.716 ± 0.089	0.723 ± 0.020	0.710 ± 0.032	0.722 ± 0.053
UpG	**1.301** ± **0.052**	**1.235** ± **0.061**	**1.281** ± **0.014**	**1.315** ± **0.027**	**1.302** ± **0.044**
CpU	0.983 ± 0.048	1.000 ± 0.064	1.108 ± 0.036	1.156 ± 0.030	1.111 ± 0.082
CpC	0.897 ± 0.050	0.822 ± 0.081	0.783 ± 0.025	0.712 ± 0.033	0.755 ± 0.082
CpA	**1.260** ± **0.074**	**1.284** ± **0.118**	**1.255** ± **0.028**	**1.344** ± **0.040**	**1.321** ± **0.069**
CpG	0.843 ± 0.065	0.850 ± 0.084	0.847 ± 0.029	0.749 ± 0.030	0.779 ± 0.064
ApU	1.064 ± 0.065	0.979 ± 0.086	0.847 ± 0.039	0.913 ± 0.030	0.940 ± 0.074
ApC	0.979 ± 0.063	0.951 ± 0.106	1.004 ± 0.026	1.113 ± 0.035	1.070 ± 0.083
ApA	1.126 ± 0.087	1.106 ± 0.089	1.120 ± 0.019	0.944 ± 0.051	0.997 ± 0.101
ApG	0.765 ± 0.055	0.903 ± 0.080	0.966 ± 0.043	0.968 ± 0.046	0.931 ± 0.088
GpU	0.953 ± 0.047	0.984 ± 0.058	1.044 ± 0.023	0.935 ± 0.024	0.948 ± 0.042
GpC	1.221 ± 0.053	1.163 ± 0.067	1.215 ± 0.024	1.201 ± 0.033	1.200 ± 0.044
GpA	0.805 ± 0.057	0.868 ± 0.064	0.842 ± 0.026	0.958 ± 0.032	0.920 ± 0.072
GpG	1.034 ± 0.057	0.978 ± 0.047	0.892 ± 0.019	0.930 ± 0.033	0.949 ± 0.055

**Note**: Dinucleotides are regarded as underrepresented (underline) or overrepresented (bold) if the relative abundance values are below 0.78 or above 1.23, respectively.

## Data Availability

The data presented in this study are available in the article and in its online supplementary material.

## Supplementary Materials

The following are available online at https://www.mdpi.com/article/10.3390/v13061044/s1. Table S1: Detailed sequence information of analyzed PRRSV strains, Table S2: *RSCU* values of ORF5 genes from analyzed PRRSV strains.
